# Zebrafish Models of Neurodevelopmental Disorders: Past, Present, and Future

**DOI:** 10.3389/fnmol.2018.00294

**Published:** 2018-08-29

**Authors:** Catalina Sakai, Sundas Ijaz, Ellen J. Hoffman

**Affiliations:** ^1^Child Study Center, Program on Neurogenetics, Yale School of Medicine, Yale University, New Haven, CT, United States; ^2^Department of Neuroscience, Yale School of Medicine, Yale University, New Haven, CT, United States

**Keywords:** zebrafish, neurodevelopmental disorders, autism spectrum disorders, epilepsy, schizophrenia, model system, genetics, neural circuits

## Abstract

Zebrafish are increasingly being utilized as a model system to investigate the function of the growing list of risk genes associated with neurodevelopmental disorders. This is due in large part to the unique features of zebrafish that make them an optimal system for this purpose, including rapid, external development of transparent embryos, which enable the direct visualization of the developing nervous system during early stages, large progenies, which provide considerable tractability for performing high-throughput pharmacological screens to identify small molecule suppressors of simple behavioral phenotypes, and ease of genetic manipulation, which has been greatly facilitated by the advent of CRISPR/Cas9 gene editing technologies. This review article focuses on studies that have harnessed these advantages of the zebrafish system for the functional analysis of genes that are strongly associated with the following neurodevelopmental disorders: autism spectrum disorders (ASD), epilepsy, intellectual disability (ID) and schizophrenia. We focus primarily on studies describing early morphological and behavioral phenotypes during embryonic and larval stages resulting from loss of risk gene function. We highlight insights into basic mechanisms of risk gene function gained from these studies as well as limitations of studies to date. Finally, we discuss advances in *in vivo* neural circuit imaging in zebrafish, which promise to transform research using the zebrafish model by illuminating novel circuit-level mechanisms with relevance to neurodevelopmental disorders.

## Introduction

In recent years, there has been growing interest in the use of zebrafish as a model system for the functional analysis of genes in neurodevelopmental disorders, which are a group of disorders characterized by alterations in behavior, cognition, communication, and/or motor function during development (American Psychiatric Association, 2013). This is due in large part to the unique features of this system, which offer distinct advantages over more traditional model systems (McCammon and Sive, 2015; Ijaz and Hoffman, 2016; Kozol et al., 2016). For example, zebrafish have transparent embryos that develop externally and rapidly, allowing for the direct visualization of neurodevelopmental processes and neural activity in an intact, functioning nervous system. In addition, zebrafish are highly tractable and produce large progenies, which facilitate the conduct of high-throughput pharmacological screens at a scale that would not be feasible in rodent models. Further, with advances in CRISPR/Cas9 gene-editing techniques in zebrafish (Hwang et al., 2013; Moreno-Mateos et al., 2015), it is now possible to generate zebrafish mutants carrying loss-of-function mutations in a gene of interest relatively rapidly and at a low cost. Given this ease of genetic manipulation, zebrafish are emerging as an optimal system for modeling the growing list of risk genes in neurodevelopmental disorders, keeping pace with the rapid rate of gene discovery in these disorders (Allen et al., 2013; Purcell et al., 2014; Sanders et al., 2015). Therefore, zebrafish have considerable potential for advancing our understanding of the roles of risk genes in the developing brain and elucidating basic biological mechanisms underlying neurodevelopmental disorders.

Despite the limitations of modeling human disorders in zebrafish, given their evolutionary divergence, several lines of evidence point to a remarkable degree of conservation, suggesting that studies in zebrafish are likely to have translational relevance to humans. First, at a structural level, zebrafish have the same major subdivisions of a vertebrate brain as mammals—forebrain, midbrain, hindbrain and spinal cord (Guo, 2009) While there are notable structural differences, such as the development of the telencephalon, which forms by a different process in zebrafish (eversion) than mammals (invagination), many brain regions in zebrafish and mammals, including the thalamus, optic tectum and cerebellum, display structural homology and are reviewed in detail in Kozol et al. (2016). In addition, early developmental genes share similar expression patterns in the brains of zebrafish and mammals, and the major neurotransmitter systems in the mammalian brain, including GABA, glutamate, dopamine, norepinephrine, serotonin, histamine and acetylcholine, are present in zebrafish (Guo, 2009). Second, there is evidence for conservation of pharmacological pathways (Burgess and Granato, 2007b; Renier et al., 2007; Rihel et al., 2010). For example, a large-scale screen of psychoactive compounds found that drugs targeting conserved neurotransmitter systems elicit similar effects on sleep in zebrafish and mammals (Rihel et al., 2010). Third, approximately 80% of risk genes associated with human disorders have an orthologous version in zebrafish, revealing considerable genetic conservation (Howe et al., 2013). Fourth, there is evidence that the neural circuits underlying basic behaviors, such as acoustic startle, prepulse inhibition, sleep and arousal, are conserved, suggesting that findings in zebrafish are likely to be relevant to our understanding of related circuits in mammals (Prober et al., 2006; Burgess and Granato, 2007a, b; Schoonheim et al., 2010; Lovett-Barron et al., 2017). These studies highlight the potential of zebrafish, with their optical transparency and amenability to whole-brain, *in vivo* imaging, for elucidating the roles of risk genes in neurodevelopmental processes and neural circuit function.

In this review article, we will focus on genetic models of the following neurodevelopmental disorders: autism spectrum disorders (ASD), intellectual disability (ID), epilepsy and schizophrenia, in zebrafish. Specifically, we will focus on early phenotypes (morphological and behavioral), rather than adult behaviors, which have been addressed in detail in other reviews (Meshalkina et al., 2018; Shams et al., 2018). We will highlight insights into basic mechanisms of risk gene function and potential drug candidates identified in zebrafish models, as well as the limitations of studies to date. Finally, we will discuss advances in functional imaging of zebrafish brain activity, which has the potential to illuminate new roles for risk genes in conserved neural circuits.

## Advances in Gene Targeting Methods in Zebrafish

Zebrafish first emerged as an optimal model system for studying vertebrate development through their use in large-scale forward genetics screens, leading to the discovery of hundreds of genes involved in early developmental processes (Granato and Nusslein-Volhard, 1996). Until recently, one of the challenges of using zebrafish as a model for reverse genetics was the limited availability of methods for generating mutants in a gene of interest. While mouse “knockouts” are generated by isolating embryonic stem cells, related methods are more challenging in zebrafish. For this reason, generating zebrafish mutants relied for a long time on Targeted Induced Local Lesions in Genomes (TILLING), which involves screening thousands of zebrafish carrying random mutations induced by the chemical N-ethyl N-nitrosourea (ENU) to identify a damaging mutation in a target gene (Moens et al., 2008). Limitations of TILLING include the time-consuming process of screening zebrafish libraries and the relatively low likelihood of identifying the desired mutation, though more recent large-scale ENU and retroviral mutagenesis projects using next-generation sequencing have improved the efficiency of this approach (Kettleborough et al., 2013; Varshney et al., 2013; Pan et al., 2015). Because zebrafish have a duplicated genome with at least two orthologs of many human risk genes that display sub-functionalization (Kozol et al., 2016), the lack of methods for rapidly generating targeted mutations greatly restricted the use of zebrafish as a genetic model.

However, the introduction of targeted nuclease technologies, including zinc finger nucleases (ZFN) and transcription activator-like effector nucleases (TALEN), transformed the field, enabling the rapid induction of damaging, heritable mutations in a gene of interest in zebrafish (Doyon et al., 2008; Meng et al., 2008; Sander et al., 2011; Dahlem et al., 2012). ZFNs and TALENs are chimeric fusion proteins designed to bind to a target site within a gene and produce double-stranded breaks that are repaired inefficiently by non-homologous end joining, resulting in insertion-deletion mutations. Despite their advantages over TILLING, a number of limitations prevented their widespread use, including challenges in predicting the efficiency of gene disruption and identifying a target site within an early exon of a gene of interest, along with the high cost of commercially available ZFNs. While TALENs improved upon many of these features, offering increased flexibility and lower cost, both methods were soon supplanted by CRISPRs.

Clustered regularly interspaced short palindromic repeats (CRISPRs) hijack an adaptive immune mechanism used by bacteria for protection against viruses (Jinek et al., 2012). To generate zebrafish mutants, a single guide RNA (sgRNA) recognizing a target genomic sequence is introduced into zebrafish embryos along with mRNA encoding the enzyme, Cas9 (Hwang et al., 2013). Following sgRNA-directed cleavage of DNA by Cas9, inefficient non-homologous end joining leads to insertion-deletion mutations, as with ZFNs and TALENs, though CRISPRs offer superior flexibility and efficiency over these earlier gene-editing methods (Hwang et al., 2013). Further, their low cost and ease of use have made this technology accessible to most laboratories, facilitating the rapid generation of zebrafish mutants and leading to a paradigm-shift in the use of zebrafish as a reverse genetics tool. While a limited number of studies to date have used ZFNs or TALENs to generate zebrafish mutants of genes associated with neurodevelopmental disorders, it is likely that a growing number of studies will harness CRISPRs for this purpose in the near future.

In contrast, the majority of zebrafish studies of neurodevelopmental disorder-associated genes to date have used morpholinos, which are modified antisense oligonucleotides that cause a transient “knockdown” of target gene expression by blocking mRNA splicing or translation (Nasevicius and Ekker, 2000; Draper et al., 2001). Given their low cost, ease of use, and until recently, the limited availability of methods for rapidly generating genetic mutants, morpholinos have been a commonly used method for analyzing gene function in early development in zebrafish. However, morpholinos have several notable drawbacks, including their transient effects, which limit the ability to investigate gene function beyond early stages, as well as their tendency to induce off-target effects (Eisen and Smith, 2008). For example, some morpholinos activate p53 via an unknown mechanism, resulting in widespread apoptosis, which may lead to nonspecific phenotypes, such as changes in head size or brain structure (Robu et al., 2007; Eisen and Smith, 2008). In addition, a growing number of studies are finding a lack of concordance between the phenotypes of genetic mutants and morpholino-induced knockdowns of the same gene, which in most cases are due to the off-target effects of morpholinos (Kok et al., 2015; Lawson, 2016). Therefore, morpholino-induced phenotypes must be interpreted with caution.

Given these limitations, early guidelines were established for confirming the specificity of morpholino-induced phenotypes, including: (i) using two morpholinos targeting distinct sites; and (ii) demonstrating rescue of the phenotype by introducing mRNA lacking the morpholino target site (Eisen and Smith, 2008). With the careful use of these controls, morpholinos have been used successfully to investigate the early developmental roles of genes in zebrafish (Eisen and Smith, 2008). However, many experiments fail to follow these guidelines (Lawson, 2016). Further, studies have found discrepancies between morphant and mutant phenotypes even when these guidelines have been followed (Stainier et al., 2017).

To address these issues, a group of leaders in the zebrafish scientific community recently established new guidelines for the use of morpholinos, requiring that all morpholino-induced phenotypes be confirmed in genetic mutants where possible, which is now feasible due to CRISPRs (Stainier et al., 2017). These guidelines further state that morpholinos may continue to be used for rapid gene “knockdown” only in cases where the phenotype under investigation has been recapitulated in a mutant (Stainier et al., 2017). While there are cases where genetic compensation has been shown to occur in mutants (Rossi et al., 2015; El-Brolosy and Stainier, 2017), the current guidelines require demonstrating the absence of the morpholino-induced phenotype in the mutant background as evidence for compensation (Stainier et al., 2017). Moving forward, following these guidelines will be critical, particularly for the interpretation of phenotypes in zebrafish models of neurodevelopmental disorders, given that morpholinos alone can induce nonspecific neural effects (Shams et al., 2018). At the same time, as mutants become the “gold standard” for reverse genetics studies in zebrafish, it will be equally important to confirm that germline mutations result in loss-of-function (Shams et al., 2018).

## Zebrafish Models of Neurodevelopmental Disorders

Here, we discuss findings from studies that used zebrafish to investigate the function of genes that are strongly associated with ASD, epilepsy, ID and schizophrenia, or to screen for the functionality of newly identified risk genes or variants (Tables 1–4). Importantly, the genetics of these disorders is complex, likely involving hundreds of risk genes, and is characterized by considerable pleiotropy, with the same genes or genomic regions conferring risk to a range of disorders (State and Šestan, 2012). For clarity, we have categorized genes in Tables 1–4 by the disorder to which they are most closely associated in the literature, noting overlapping associations where applicable, though studies of the biological functions of these genes are likely to be relevant across diagnostic boundaries. While zebrafish have also been used to study other neurodevelopmental disorders, including attention-deficit/hyperactivity disorder (ADHD; Lange et al., 2012a,b), Bardet-Biedl syndrome (BBS; Zaghloul et al., 2010; Heon et al., 2016; Lindstrand et al., 2016), and maple syrup urine disease (MSUD; Friedrich et al., 2012), we focus on ASD, epilepsy, ID and schizophrenia, which have been the subject of most zebrafish studies to date and highlight the advantages of this system for the functional analysis of risk genes. Moreover, while an increasing number of studies are investigating complex behaviors in adult zebrafish, such as social behaviors, it is important to observe that there are limitations of face validity, such that it is not possible to recapitulate fully the symptoms of neurodevelopmental disorders in zebrafish (or any animal model), and this is not a prerequisite for demonstrating the relevance of the model. Therefore, in this review article, we focus our discussion on embryonic and larval phenotypes, which highlight the unique strengths of the zebrafish system for illuminating conserved roles of risk genes in basic biological pathways and brain circuits underlying simple behaviors, which are likely to have translational relevance.

**Table 1:** Zebrafish models of autism spectrum disorders (ASD)-associated genes and genetic syndromes.

Human Gene(s)	Zebrafish Gene(s)	Disorder*	Method**	Phenotype(s)	Rescue	Reference
***CHD7***	***chd7***	CHARGE	*MO*	Abnormal somite segmentation (13 somite) Small eyes, pericardial edema, abnormal otoliths (48 hpf) Abnormal vascular patterning (48 hpf), cranial neural crest (34 –36 hpf), cranial neuron (48 hpf), and retinal (72 hpf) development; decreased bone mineralization (8 dpf, 14 dpf)	Zebrafish mRNA rescues eye, heart, and otolith abnormalities	Patten et al. (2012)
			*MO*	Developmental defects as in Patten et al. (2012) Decreased cell proliferation (25 hpf) Abnormal craniofacial cartilage (4 dpf)	*fbxl10* MO reduced developmental defects, cell proliferation deficit	Balow et al. (2013)
			*MO* **CRISPR Mutant**	***chd7 −/− mutants and chd7 MO:*** - Pericardial edema, cardiomegaly (3 dpf); abnormal pigmentation (4 dpf) - Reduction of vagal innervation of GI tract in foregut more than hindgut (5 dpf) - Decreased GI emptying (5 dpf)	Prokinetic drugs do not rescue GI emptying	Cloney et al. (2018)
***CHD8***	***chd8***	ASD	*MO*; *F0* *CRISPR*	Macrocephaly (increased interorbital/intertectal distance (4.3 dpf) in MO; increased interorbital distance in F0 CRISPR) Developmental marker expression changes: expansion of *chordin* (shield stage); increased mid/forebrain neuronal progenitor marker *otx2* (tail bud) and *dlx2* in prethalamus (24 hpf; MO) Decreased GI motility (MO) and # of enteric neurons (6 dpf; MO and F0 CRISPR)	−	Bernier et al. (2014)
			*MO*	Ectopic HuC/D expression in anterior forebrain (2 dpf) Increased cell proliferation in brain (2 dpf)	−	Sugathan et al. (2014)
***CNTNAP2***	***cntnap2a/b***	ASD EP CDFE	**ZFN** **Mutant**	Decreased forebrain GABAergic neurons (4 dpf) Increased sensitivity to drug-induced seizures; nighttime hyperactivity (4–6 dpf) GABA-modifying drugs induce differential behavioral responses Increased sensitivity to behavioral activation by NMDA antagonists	Estrogenic compounds identified in drug screen rescue nighttime hyperactivity	Hoffman et al. (2016)
***DYRK1A***	***dyrk1aa***	ASD	**TALEN** **Mutant**	Normal development, morphology (24–48 hpf); normal larval locomotor activity (5–7 dpf) No difference in brain size (2 weeks old); increased apoptosis in brain (3 weeks old) *Adult Phenotypes:* Microcephaly; decreased anxiety, social behavior impairments, decreased *c-fos*, *crh* expression in hypothalamic regions after social interaction, social isolation, respectively	−	Kim et al. (2017)
***FMR1***	***fmr1***	FXS	*MO* *OE*	***fmr1 MO:*** - Abnormal mid-hindbrain boundary, altered *dlx2a* expression (24 hpf) - Increased branching of trigeminal and Rohon-Beard neurites (24 hpf) - Axon defasciculation of lateral longitudinal fasciculus (5 dpf) - Fewer trigeminal neurons; craniofacial abnormalities (5 dpf)	*fmr1* mRNA rescues *dlx2a* expression; MPEP rescues neurite branching, number of trigeminal neurons	Tucker et al. (2006)
				***OE fmr1 mRNA:*** - Decreased branching of trigeminal and Rohon-Beard neurites (24 hpf) - Increased trigeminal neurons		
			**TILLING** **Mutant**	No gross morphological abnormalities; no differences in *dlx2a* expression No craniofacial abnormalities (5 dpf); no defects in Rohon-Beard neurite branching	−	den Broeder et al. (2009)
***MECP2***	***mecp2***	RTT	**TILLING** **Mutant**	Increased contractions during coiling events (25 hpf) Increased C-bend duration during escape response (51 hpf) Decreased locomotor activity and thigmotaxis (6 dpf)	−	Pietri et al. (2013)
***MECP2***	***mecp2***	RTT	*MO*; *F0* *CRISPR*; *OE*	***mecp2 MO:*** - Increased neural, glial proliferation; decreased differentiation (48 hpf) (MO and F0 CRISPR) - Upregulation of Notch signaling genes; increased brain *her2*, *id1* expression (48 hpf) ***mecp2 OE:*** - Decreased cell proliferation, increased neuronal differentiation (48 hpf) - Decreased *id1* expression (48 hpf)	*mecp2* mRNA, *her2* MO, *id1* MO rescue proliferation, differentiation phenotypes in *mecp2* MO; *id1* MO rescues *her2* levels in *mecp2* MO	Gao et al. (2015)
			*MO;* **TILLING** **Mutant**	***mecp2*−/− *mutant and mecp2 MO:*** - Decreased trigeminal neurite length (24 hpf); less severe in mutant - Downregulation of *sema3f*, *sema5b* and *robo2* (24 hpf); less severe in mutant - MO + mutant does not worsen mutant phenotypes ***mecp2 MO:*** - Increased apoptosis (16 hpf, 24 hpf); delayed response to tactile stimuli (48 hpf)	Zebrafish *mecp2* mRNA rescues neurite length; *HuC-mecp2*, *sema5b*, *robo2* mRNA rescue neurite length and tactile response phenotypes	Leong et al. (2015)
			*MO*	Decreased Rohon-Beard axon length/branching (28 hpf) Motor neuron axon abnormalities: caudal primary (28 hpf), caudal secondary (72 hpf) Decreased touch response (28 hpf, 72 hpf) Increased *bdnf* expression (24 hpf) Increased presynaptic SV2-stained area, abnormal formation of neuromuscular junction (72 hpf; also in *bdnf OE*); *bdnf* MO decreases presynaptic area in *mecp2* MO	Human *MECP2* mRNA rescues motor axon, touch response phenotypes; *MECP2* lacking MBD domain does not rescue	Nozawa et al. (2017)
			**TILLING** **Mutant**	Body length: decreased at 2 dpf; normal at 7 dpf GI tract discoloration, abnormal droplets (4 dpf, 7 dpf) Expression of inflammatory marker *crp:* normal at 3 dpf; increased at 4–5 dpf	*mecp2* OE partially rescues *tnfa* expression (24 hpf); *tnfa* OE does not rescue body length, GI-associated neutrophil number (2–3 dpf)	van der Vaart et al. (2017)
				Neutrophil number: normal at 3 dpf, increased at 4–5 dpf; increased in GI tract (2–5 dpf) Dysregulated cytokine expression; decreased *tnfa* expression (6 hpf, 24 hpf–7 dpf) No *tnfa* activation during acute inflammation (3 dpf)		
***SHANK3***	***shank3a***	ASD PMS	*MO*	Abnormal mid-hindbrain boundary, ventricle size (28–30 hpf) Increased apoptosis in CNS (reversed by p53 MO) (24–28 hpf) Decreased GABAergic neurons (mid/hindbrain), glutamatergic neurons (hindbrain; 48 hpf) Abnormal escape responses and seizure-like behaviors (72 hpf)	Human *SHANK3* mRNA partially rescues apoptosis	Kozol et al. (2015)
	***shank3b***		**CRISPR** **Mutant**	Transient developmental delay (24 hpf) Decreased HuC:RFP levels (24–72 hpf), though difference decreases over time Decreased locomotor activity (7 dpf) *Adult Phenotypes:* Increased brain size, decreased locomotor activity/thigmotaxis, social behavior impairments, repetitive behaviors, decreased brain homer1, synaptophysin levels	−	Liu et al. (2018)
***SYNGAP1***	***syngap1b***	ASD	*MO*	Abnormal mid-hindbrain boundary, ventricle size (28–30 hpf) Increased apoptosis in CNS (not reversed by p53 MO) (24–28 hpf) Microcephaly, developmental delay (48–72 hpf) Decreased GABAergic neurons in midbrain, glutamatergic neurons in hindbrain (48 hpf) Abnormal escape responses and seizure-like behaviors (72 hpf)	Human *SYNGAP1* mRNA partially rescues apoptosis	Kozol et al. (2015)
***TSC1***	***tsc1a***	TSC	*MO*	Body curvature, cysts in tubular/glomerular regions of pronephros Decreased left-right asymmetry; increased TOR activity (24 hpf), ciliary length (20–24 hpf)	−	DiBella et al. (2009)
***TSC2***	***tsc2***	TSC	**TILLING** **Mutant**; **OE**; **Cell** **transplantation**	***tsc2*−/−:** - Deflated swim bladder, enlarged liver, increased TORC1 activity (7 dpf); death by 11 dpf - Increased size of hepatocytes (9 dpf), brain and spinal cord neurons (7.5 dpf) - Forebrain gray and white matter disorganization, ectopic neurons in white matter (7.5 dpf) ***Mutant tsc2 mRNA OE:*** - Increased TORC1 activity (10–11 hpf); dorsal expansion of hindbrain (27 hpf) ***Cell transplantation of tsc2−/− cells into wild-type at 4 hpf:*** - Increased TORC1 activity in mutant, not wild-type, cells (7.5 dpf; adult) - Disrupted gray-white matter boundaries (7.5 dpf; adult) - Ectopic wild-type cells in white matter in some chimeric embryos (7.5 dpf) - Mutant cell clusters found in gray-white matter boundary, gray matter (adult)	Zebrafish mRNA rescues elevated TORC1 activity (partial rescue by human mRNA); rapamycin reverses elevated TORC1 activity	Kim et al. (2011)

**Key:** hpf, hours post fertilization; dpf, days post fertilization; *Disorder: ASD, autism spectrum disorder; CDFE, cortical dysplasia focal epilepsy; CHARGE, CHARGE Syndrome; EP, epilepsy; FXS, Fragile X syndrome; PMS, Phelan-McDermid Syndrome; RTT, Rett syndrome; TSC, tuberous sclerosis complex; **Methods of Risk Gene Disruption: CRISPR, clustered regularly interspaced short palindromic repeats; CRISPR F0, Mosaic CRISPR-injected embryo; MO, Morpholino; OE, overexpression; TALEN, transcription activator-like effector nuclease; TILLING, targeted induced local lesions in genomes; ZFN, zinc finger nuclease.

**Table 2:** Zebrafish models of genes in 16p11.2 interval.

Human Gene(s)	Zebrafish Gene(s)	Disorder*	Method**	Phenotype(s)	Rescue	Reference
**21 genes^#^**	**22 orthologs^#^**	ASD SCZ ID	*MO*	Brain or eye abnormalities at 24 hpf (20/22 genes) Muscle and tail abnormalities at 24 hpf for most genes Decreased spontaneous movement at 24 hpf (7 genes), touch response at 48 hpf (14 genes) Abnormal axon tract development at 36 hpf (6 genes); altered pigmentation (8 genes)	Human or zebrafish mRNA rescue phenotypes for all genes (except *mvp*)	Blaker-Lee et al. (2012)
**29 genes^##^**	***kctd13***	ASD SCZ ID	*OE* (Human mRNA in zebrafish); MO *(kctd13)*	*KCTD13* is only human mRNA where OE causes head size PT ***Human KCTD13 mRNA OE in zebrafish*:** - Microcephaly; fewer neurons in telencephalon (4.5 dpf) - Increased apoptosis in brain (3 dpf) - Decreased cell proliferation in brain (2 dpf) ***kctd13 MO:*** - Macrocephaly; increased neurons in telencephalon (4.5 dpf) - Increased cell proliferation in brain (2 dpf) Mouse *Kctd13* knockdown *in vitro*/*in vivo*: Increased cell proliferation	Human mRNA + *kcdt13* MO rescue head size phenotypes	Golzio et al. (2012)
***KCTD13***	***kctd13***	−	**CRISPR** **Mutant**	***Zebrafish and mouse KCTD13 mutants:*** - No changes in head size or cell proliferation (4.5 dpf) - Increased RhoA in brain (adult zebrafish; mice ≥ P18) Decreased synaptic transmission in mice	RhoA inhibitor rescues synaptic transmission defects in mice	Escamilla et al. (2017)
***DOC2A*** ***FAM57B***	***doc2a*** ***fam57ba***	−	*MO*; **TALEN** **Mutant**	Genetic interactions between 16 pairs of genes in 16p11.2 interval, including *fam57ba* and *doc2a*, in MO screen for brain/ventricle morphology phenotypes at 24 hpf ***doc2a*^+/−^*fam57ba*^+/−^:** - Hyperactivity and seizure sensitivity (7 dpf) - Increased body length and some head size dimensions (12 dpf) ***fam57ba*^−/–^:** - Hyperactivity and seizure sensitivity (7 dpf), less than in double heterozygotes - Strong increase in body length and head size (12 dpf) - Increased lipid content	Valproic acid and carbamazepine rescue drug-induced seizures	McCammon et al. (2017)

**Key:** hpf, hours post fertilization; dpf, days post fertilization; *Disorder: ASD, autism spectrum disorder; EP, Epilepsy; ID, intellectual disability; SCZ, schizophrenia; **Methods of Risk Gene Disruption: CRISPR, clustered regularly interspaced short palindromic repeats; MO, Morpholino; OE, overexpression; TALEN, transcription activator-like effector nuclease; **^#^Human Genes in 16p11.2 interval in Blaker-Lee et al. (2012):** ALDOA, ASPHD1, C16orf53, CDIPT, CORO1A, DOC2A, FAM57B, GDPD3, HIRIP3, INO80E, KCTD13, KIF22, MAPK3, MAZ, MVP, PPP4C, PRRT2, SEZ6L2, TAOK2, TBX6, YPEL3; **Zebrafish Orthologs:** aldoaa, asphd1, c16orf53, cdipt, coro1a, doc2a, fam57ba, gdpd3, hirip3, ino80e, kctd13, kif22, mapk3, maz, mvp, ppp4ca, prrt2, sez6l2, taok2a, taok2b, tbx24, ypel3; **^##^Human Genes in 16p11.2 interval in Golzio et al. (2012):** ALDOA, ASPHD1, BOLA2, C16orf53, C16orf54, C16orf92, CDIPT, CORO1A, DOC2A, FAM57B, GDPD3, HIRIP3, INO80E, GIYD2, KCTD13, MAPK3, MAZ, MVP, PPP4C, PRRT2, QPRT, SEZ6L2, SPN, SULT1A3, SULT1A4, TAOK2, TBX6, TMEM219, YPEL3.

**Table 3:** Zebrafish models of epilepsy and intellectual disability-associated genes.

Human Gene(s)	Zebrafish Gene(s)	Disorder*	Method**	Phenotype(s)	Rescue	Reference
***CHD7***	***chd7***	DS/ID (* in this study) ASD	*MO*	Morphological abnormalities: pericardial edema, microcephaly, body curvature, no swim bladder, decreased growth (≥48 hpf) Abnormal movement (4 dpf) Epileptiform discharges in tectal field-potential recordings (4 dpf)	−	Suls et al. (2013)
***PK1***	***pk1a***	EP	*OE*	Gastrulation defects: Reduced anterior-posterior length (20 hpf); lateral expansion of somites (8–10 somite stage) OE zebrafish *pk1a* mRNA with human mutation (R104Q) causes less severe defects	−	Bassuk et al. (2008)
			*MO*	Increased sensitivity to drug-induced seizures (48 hpf) Retinal inner plexiform layer defects (76–78 hpf) Normal visual startle response (5 dpf)	Zebrafish mRNA partially rescues retinal defects; mRNA with human R150H, Y465H mutations do not	Mei et al. (2013)
***PSMD12***	***psmd12***	ID	*F0* *CRISPR*	Decreased size of optic tecta (3 dpf) Proximal renal tubule defects, decreased proximal tubule convolution area (4 dpf) Craniofacial abnormalities (3 dpf)	−	Kury et al. (2017)
***RHEB***	***rheb***	ID	*F0* *CRISPR;* *OE*	***F0 CRISPR:*** - Microcephaly (decreased head area excluding eyes; 5 dpf) ***OE human RHEB and RHEB carrying human mutations:*** - Macrocephaly (5 dpf)	Rapamycin rescues macrocephaly	Reijnders et al. (2017)
***SCN1A***	***scn1lab***	DS EP ASD	**ENU** **Mutant;** *MO*	Abnormal optokinetic response (OKR; 5 dpf; MO and mutant) Darkened pigmentation (MO and mutant) Death by 14 dpf (mutant)	Stimulation of hindbrain saccade generator rescues OKR (morphants)	Schoonheim et al. (2010)
			**ENU** **Mutant**	Spontaneous seizure-like activity Abnormal forebrain electrographic activity (3–7dpf)	Clemizole identified in drug screen rescues seizure activity	Baraban et al. (2013)
			*MO*	Hyperactivity (3–5 dpf); spontaneous electrographic activity (5 dpf) Increased sensitivity to hyperthermia (5–7 dpf)	Fenfluramine and valproate rescue seizure activity	Zhang et al. (2015)
			**ENU** **Mutant**	Seizure activity	Fenfluramine and dimethadione rescue seizure activity	Dinday and Baraban (2015)
				Lower levels of serotonin in *scn1lab***−/−** head homogenates at 7 dpf Zebrafish express orthologs of human serotonin (5HT) receptor subtypes at 5 dpf	5HT_1D_, 5HT_2C_, 5HT_2A_-agonists, fenfluramine rescue seizure activity	Sourbron et al. (2016)
				Nighttime hyperactivity (5 dpf) Open field: Increased thigmotaxis, decreased movement (5 dpf) No differences in GABAergic neurons (5 dpf) 5-HT_2A_ (*htr2aa/b*) and 5-HT_2C_ (*htr2cl1*) orthologs expressed in larval heads in wild-type and *scn1lab***−/−** mutants and in adult wild-type brains Clemizole binds to serotonin receptors, 5HT_2A_ and 5HT_2B_, in radioligand binding assay	Clemizole, diazepam rescue nighttime hyperactivity, thigmotaxis Trazodone and lorcaserin identified in screen of 5HT compounds rescue seizure activity	Grone et al. (2017) Griffin et al. (2017)
***STXBP1***	***stxbp1a/b***	EP ID	**CRISPR** **Mutant**	***stxbp1a*−/−:** Abnormal craniofacial development; failure to hatch; death by 10 dpf Darkened pigmentation Immobility; no response to dark flash (5–6 dpf) No spontaneous seizure-like behaviors or electrographic seizures Reduced heart rate and metabolism ***stxbp1b*−/−:** Darkened pigmentation No spontaneous seizure-like behaviors Electrographic seizure events in forebrain field potential recordings Reduced response to dark flash (5dpf)	−	Grone et al. (2016)
***STX1B***	***stx1b***	EP	*MO*	No swim bladder, pericardial edema, tail curvature (5 dpf) Lack of touch response in ~40% of embryos (4 dpf) Abnormal behaviors: repetitive fin, increased orofacial, myoclonic-like movement (5 dpf) Spontaneous epileptiform activity on local field potentials of optic tecta: polyspiking discharges, high frequency oscillations (5 dpf)	CNS-specific expression of human mRNA rescues epileptiform activity; mRNA with a human mutation (V216E) does not rescue	Schubert et al. (2014)
***TRAPPC6B***	***trappc6b***	EP ASD	*MO*	Microcephaly (2 dpf, 5 dpf) Increased apoptosis (24 hpf) Decreased baseline activity (5 dpf) Increased sensitivity to drug-induced seizures Increased neuronal activity at baseline and in response to seizure-inducing drug (5 dpf)	Human mRNA rescues motility deficit	Marin-Valencia et al. (2018)

**Key:** hpf, hours post fertilization; dpf, days post fertilization; *Disorder: ASD, autism spectrum disorder; DS, Dravet syndrome; EP, Epilepsy; ID, intellectual disability; **Methods of Risk Gene Disruption: CRISPR, clustered regularly interspaced short palindromic repeats; CRISPR F0, Mosaic CRISPR-injected embryo; ENU, N-ethyl N-nitrosourea-induced mutant; MO, Morpholino; OE, overexpression.

**Table 4:** Zebrafish models of schizophrenia-associated genes.

Human Gene(s)	Zebrafish Gene(s)	Disorder	Method**	Phenotype(s)	Rescue	Reference
***CNTN4*** ***FURIN*** ***TSNARE1***	***cntn4*** ***furina*** ***tsnare1***	SCZ	*MO*; *F0* *CRISPR;* *OE*	***furina MO:*** - Microcephaly (forebrain-midbrain region; 3 dpf; MO and F0 *CRISPR*) - Decreased cell proliferation - Increased apoptosis ***tsnare/cntn4 OE:*** - Microcephaly (forebrain-midbrain region; 3 dpf) - Increased cell proliferation - Increased apoptosis	Human *FURIN* mRNA rescues microcephaly and cell proliferation in *furina* morphant	Fromer et al. (2016)
***DISC1***	***disc1***	SCZ	*MO;* ***TILLING*** ***Mutant***	***disc1*−/− mutant and disc1 MO:** - Abnormal brain morphology, small brain ventricles, abnormal muscle segments, bent tail (24 hpf); disorganized, decreased/missing axon tracts (36 hpf) - Abnormal pigmentation, bent tail, swim bladder does not inflate (5 dpf, mutant) Evidence that Wnt signaling pathway underlies phenotypes due to loss of *disc1* in MO: - *DISC1* mRNA lacking GSK3β domain does not rescue brain or muscle phenotypes - β-catenin activation, dominant negative-GSK3β rescue forebrain, axon phenotypes - Activation of noncanonical Wnt pathway rescues muscle/tail phenotypes	Human *DISC1* mRNA rescues abnormal morphology in mutant (24 hpf); CNS-specific zebrafish *disc1* mRNA rescues brain, axon, not tail phenotypes in MO	De Rienzo et al. (2011)
			*MO*	Abnormal brain/ventricle morphology, abnormal muscle segments, bent tail, disorganized axon tracts (24 hpf)	Human *DISC1* mRNA rescues; mRNA with human mutations either fully (S704C), partially (L607F), or do not (R264Q) rescue	Singh et al. (2011)
			***TILLING*** ***Mutant***	Changes in expression of *rx3*, marker of hypothalamic neuron precursors (24 hpf–5dpf) Increased cell proliferation in hypothalamus at 24 hpf, decreased at 3 dpf Abnormal numbers of neuroendocrine neurons in hypothalamus (2–3 dpf, 5 dpf) No change in shoal cohesion after alarm substance or osmotic stress (5 dpf) *Adult phenotypes:* Increased freezing (open field); no light-dark preference; no increase in bottom dwell time after alarm substance	−	Eachus et al. (2017)
***MAPK3***	***mapk3***	SCZ	*MO*	Microcephaly (forebrain-midbrain region; 4 dpf) Decreased cell proliferation (72 hpf)	Human *KCTD13* OE rescues microcephaly, cell proliferation in *mapk3* MO	Gusev et al. (2018)
***SHANK3***	***shank3a/b***	SCZ (*in this study) ASD PMS	*MO*	Microcephaly Abnormal larval escape responses	Rat mRNA partially rescues escape response; Human *de novo* R1117X mutation does not; R536W partially rescues	Gauthier et al. (2010)

**Key:** hpf, hours post fertilization; dpf, days post fertilization; *Disorder: ASD, autism spectrum disorder; SCZ, schizophrenia; PMS, Phelan-McDermid Syndrome; **Methods of Risk Gene Disruption: CRISPR F0, Mosaic CRISPR-injected embryo; MO, Morpholino; OE, overexpression; TILLING, targeted induced local lesions in genomes.

### Autism Spectrum Disorder

ASDs are a devastating group of neurodevelopmental disorders characterized by marked impairments in social behavior and communication, and by the presence of restricted, repetitive behaviors (American Psychiatric Association, 2013). In recent years, large-scale, whole-exome sequencing studies have led to a rapidly expanding list of reliable, “high confidence” ASD risk genes, which are beginning to reveal common biological mechanisms (Willsey et al., 2013; De Rubeis et al., 2014; Iossifov et al., 2014; Sanders et al., 2015). Despite this progress, how the disruption of these genes leads to the alteration of specific cell types and neural pathways during early stages of brain development remains poorly understood. A growing number of studies have used zebrafish models to investigate the function of “high confidence” ASD risk genes and genes linked to ASD-associated syndromes, such as Fragile X syndrome, tuberous sclerosis complex, Rett syndrome, and CHARGE syndrome (Table 1), as well as genes found in the 16p11.2 chromosomal interval (Table 2), where copy number variants (CNVs) have been associated with ASD, ID and schizophrenia (Kumar et al., 2008; Marshall et al., 2008; Weiss et al., 2008; McCarthy et al., 2009).

Recent studies of zebrafish models of ASD risk genes are beginning to shed light on relevant neurobiological mechanisms. For example, because excitatory-inhibitory imbalance has been implicated as a potential mechanism underlying ASD (and epilepsy; Rubenstein and Merzenich, 2003), some studies have investigated the extent to which disruption of ASD risk genes alters inhibitory GABAergic and excitatory glutamatergic neurons during early brain development using transgenic lines that label these cell populations. For example, Kozol et al. (2015) found that knockdown of two of the zebrafish orthologs of the ASD-associated genes, *SHANK3* and *SYNGAP1*, led to fewer GABAergic neurons in the midbrain (and hindbrain for *shank3a*) and glutamatergic neurons in the hindbrain. In addition, our group found that zebrafish mutants of both orthologs of the ASD- and epilepsy-linked gene, *CNTNAP2*, display deficits in forebrain GABAergic neurons, but lack regional deficits in glutamatergic neurons (Hoffman et al., 2016). Interestingly, GABAergic deficits were also found in mouse knockouts of *Cntnap2* (Penagarikano et al., 2011), suggesting that this gene affects conserved pathways in fish and mice.

In addition, given that differences in head and brain size, particularly macrocephaly, and changes in neuron number or organization have been described in ASDs (Courchesne et al., 2011; Stoner et al., 2014), a number of morpholino-based studies have examined the effects of decreased ASD risk gene expression on related phenotypes. For example, reduced expression of most zebrafish orthologs of genes in the 16p11.2 chromosomal interval led to structural brain abnormalities at 24 h post fertilization (hpf), including smaller brain ventricles, altered midbrain-hindbrain boundary, or a straight midbrain (Blaker-Lee et al., 2012). In addition, reduced expression of *shank3a* and *syngap1b*, led to alterations in the midbrain-hindbrain boundary, ventricle size, and microcephaly (Kozol et al., 2015). Further, knockdown of the “high confidence” ASD risk gene, *CHD8*, was associated with macrocephaly and increased cell proliferation in the brain (Bernier et al., 2014; Sugathan et al., 2014). However, because morpholinos themselves can cause nonspecific neural phenotypes, confirmation of these phenotypes in germline mutants is a critical next step.

Several studies have also investigated behavioral phenotypes in zebrafish larvae as a means of elucidating how risk gene disruption leads to alterations in simple behaviors. For example, zebrafish mutants of *MECP2*, the gene responsible for Rett syndrome, display decreased locomotor activity, reduced thigmotaxis (wall preference), and longer touch-evoked escape responses, indicating that loss of *mecp2* affects embryonic and larval behaviors (Pietri et al., 2013). In addition, *shank3a* and *syngap1b* morphant larvae display abnormal escape responses (Kozol et al., 2015), while *shank3b* mutant larvae (lacking the function of the other zebrafish ortholog of *SHANK3*) exhibit reduced locomotor activity (Liu et al., 2018). Mutant larvae of *scn1lab*, an ortholog of the ASD- and epilepsy-associated genes, *SCN1A* and *SCN2A*, exhibit spontaneous seizures (discussed in the “Epilepsy” section), as well as nighttime hyperactivity and increased thigmotaxis (Grone et al., 2017). *cntnap2ab* mutants also display nighttime hyperactivity and increased sensitivity to drug-induced seizures (Hoffman et al., 2016). Further, double heterozygous mutants of two genes in the 16p11.2 interval, *doc2a* and *fam57ba*, show greater hyperactivity and drug-induced seizure sensitivity than single homozygous mutants of each gene, suggesting a genetic interaction (McCammon et al., 2017).

An important advantage of studying larval behavioral phenotypes is their amenability to high-throughput quantitative assays and small molecule screens (Prober et al., 2006; Kokel et al., 2010; Rihel et al., 2010). We capitalized on this approach to investigate rest-wake activity in zebrafish *cntnap2ab* mutants, which display nighttime hyperactivity (Hoffman et al., 2016). By comparing the behavioral “fingerprint” of *cntnap2ab* mutants across a range of rest-wake behavioral parameters with a database of the responses of wild-type fish to over 550 psychoactive compounds (Rihel et al., 2010), we predicted compounds that might rescue the mutant behavioral phenotype and tested a select group of these compounds to identify suppressors (Hoffman et al., 2016). Intriguingly, we found that estrogenic compounds selectively suppress nighttime hyperactivity in *cntnap2ab* mutants, revealing a new neurochemical pathway not previously associated with this gene (Hoffman et al., 2016). In this way, pharmaco-behavioral profiling of zebrafish ASD risk gene mutants represent a promising first-pass screening approach to identify potential pharmacological candidates for further investigation.

Zebrafish mutants also provide an opportunity to investigate risk gene function over the course of development from embryonic stages through adulthood. This is particularly relevant for ASD, where many risk genes are highly expressed in the human brain during embryonic and fetal stages (State and Šestan, 2012). Interestingly, two recent studies found that zebrafish mutants of ASD risk genes display distinct phenotypes at different developmental stages. First, zebrafish mutants of one ortholog of *DYRK1A*, a “high confidence” ASD risk gene located in the Down syndrome critical region, developed normally with no gross morphological or locomotor abnormalities during embryonic and larval stages, yet displayed increased apoptosis in the brain at 3 weeks old, and microcephaly and behavioral abnormalities in adulthood (Kim et al., 2017). Second, mutants of *shank3b*, the second ortholog of *SHANK3*, exhibited transient developmental delay at 24 hpf and fewer CNS neurons at 24–72 hpf, yet this difference diminished over time (Liu et al., 2018). In contrast, adult *shank3b* mutants display increased brain size and behavioral deficits (Liu et al., 2018). These studies highlight the importance of assessing phenotypes along a developmental trajectory.

With regard to ASD-associated syndromes, several studies have investigated signaling pathways in zebrafish models of Rett syndrome. For example, one study found that *mecp2* knockdown led to increased proliferation of neural precursors and decreased neuronal differentiation, which were reversed by simultaneously knocking down *id1* or *her2*, implicating Id1-HER2 signaling as a downstream pathway (Gao et al., 2015). Another study tracked inflammatory phenotypes in *mecp2* mutants over the course of development, finding decreased expression of the proinflammatory cytokine, *tnfa*, as early as 6 hpf, while differences in other cytokines emerge later (van der Vaart et al., 2017). This study further highlights the relevance of assessing phenotypes along a developmental trajectory. Reduction of *mecp2* expression was also associated with abnormalities in both sensory and motor axon outgrowth (Leong et al., 2015; Nozawa et al., 2017). In addition, (Leong et al., 2015) found that the axon guidance molecules, *sema5b* and *robo2*, are downregulated in *mecp2* morphants and mutants, and that co-expression of these genes in morphants rescues decreased trigeminal neurite length and delayed touch responses. Of note, the structural and gene expression phenotypes were more severe in morphants than mutants, but were not worsened by the introduction of the morpholino in mutant embryos, suggesting there may be compensation (Leong et al., 2015). This underscores the importance of directly comparing mutant and morphant phenotypes.

However, zebrafish models of Fragile X syndrome provide a cautionary tale regarding the off-target effects of morpholinos. That is, morpholino-induced knockdown of *fmr1* was associated with multiple neurodevelopmental phenotypes, including alterations in the midbrain-hindbrain boundary, abnormal neurite branching, and craniofacial abnormalities (Tucker et al., 2006). However, none of these phenotypes was replicated in two lines of *fmr1* mutants, which lacked Fmr protein expression by western blot (den Broeder et al., 2009). *KCTD13* offers another example where morphant phenotypes did not replicate in a mutant. *KCTD13* is found in the 16p11.2 chromosomal interval, where deletions are associated with macrocephaly, ASD and ID, and duplications with microcephaly, ASD and schizophrenia. Consistent with this association, Golzio et al. (2012) found that morpholino-induced *kctd13* knockdown and overexpression of human *KCTD13* led to reciprocal phenotypes of macrocephaly and microcephaly, respectively, as well as related changes in cell proliferation in the brains of zebrafish larvae, implicating this gene as a potential driver of head size phenotypes (Golzio et al., 2012). However, a recent study did not identify differences in head size or cell proliferation in zebrafish (or mouse) mutants of *KCTD13* (Escamilla et al., 2017). These studies underscore the importance of validating morpholino-induced phenotypes in genetic mutants.

Zebrafish models of ASD-associated syndromes also provide evidence for the conservation of molecular pathways. For example, both mutants and morphants of *CHD7*, the gene that is associated with most cases of CHARGE syndrome, display pericardial edema and cardiac abnormalities, consistent with cardiac abnormalities found in affected individuals (Patten et al., 2012; Balow et al., 2013; Cloney et al., 2018). In addition, *chd7* mutants (and morphants) display decreased GI emptying (Cloney et al., 2018), while *chd8* morphants showed reduced GI motility (Bernier et al., 2014), which may be relevant to GI symptoms in individuals carrying mutations in these genes. Further, zebrafish models of tuberous sclerosis complex, including *tsc1a* morphants and *tsc2* mutants, display increased TORC activity (DiBella et al., 2009; Kim et al., 2011). Consistent with findings in mammals, rapamycin reverses elevated TORC1 activity in *tsc2* mutants (Kim et al., 2011). Interestingly, by transplanting cells from *tsc2* to wild-type embryos at the blastula stage, Kim et al. (2011) showed that increased TORC1 activity is cell autonomous, but that mutant cells also induce non-cell autonomous effects, leading to the ectopic localization of wild-type cells in the white matter. Transplanted mutant cells were also found in abnormal clusters at the gray-white matter boundary in adult brains, suggestive of brain hamartomas found in individuals with this disorder (Kim et al., 2011). Together, these studies highlight the potential of zebrafish models of ASD risk genes to reveal conserved pathways with translational relevance to mammals.

### Epilepsy

Epilepsy is a common neurological condition characterized by recurrent seizures (Myers and Mefford, 2015). There has been considerable progress in risk gene discovery in epilepsy from studies of Mendelian syndromes in large family pedigrees, as well as through the identification of *de novo* single nucleotide variants and CNVs in affected individuals (Hildebrand et al., 2013). Here, we highlight studies using zebrafish models of genetic epilepsy syndromes (Table 3). Zebrafish offer several advantages in this regard. First, zebrafish larvae display robust, seizure-like behaviors, including rapid burst-like and circling movements, following exposure to the GABA-A antagonist, pentylenetetrazol (PTZ; Baraban et al., 2005), providing a quantifiable readout of seizure susceptibility. That is, increased sensitivity to PTZ-induced seizures has been shown in zebrafish models of epilepsy and ASD (Mei et al., 2013; Hoffman et al., 2016; McCammon et al., 2017). Second, zebrafish mutants of epilepsy-associated genes have been shown to exhibit spontaneous seizures as larvae (Baraban et al., 2013; Grone et al., 2016). Third, both drug-induced and spontaneous locomotor seizures are associated with electrographic seizures (Baraban et al., 2005, 2013), and are readily quantifiable in high-throughput assays (Baraban et al., 2013; Hong et al., 2016; Fuller et al., 2018), making zebrafish an optimal model for drug discovery in epilepsy syndromes.

In particular, zebrafish mutants of *scn1lab* have been used as a model of Dravet syndrome, a severe, intractable form of epilepsy, which in most cases is caused by mutations in *SCN1A* (Baraban et al., 2013). Homozygous *scn1lab* mutants display spontaneous seizures beginning at 4 days post fertilization (dpf), as well as electrographic seizures in forebrain extracellular field recordings, which worsen from 3–7 dpf (Baraban et al., 2013). Interestingly, *scn1lab* mutants were first identified in a forward genetic screen of ENU-mutagenized fish due to their inability to sustain saccadic eye movements during the optokinetic response (OKR; Schoonheim et al., 2010). These mutants have an abnormal pigmentation pattern and die by 14 dpf (Schoonheim et al., 2010). Using these mutants, Baraban et al. (2013) performed a high-throughput screen of 320 compounds, and identified clemizole, a U.S. Food and Drug Administration-approved drug and antihistamine, as a suppressor of both seizure-like behaviors and electrographic seizures. A subsequent study found that clemizole has activity at 5-HT_2A_ and 5-HT_2B_ receptors in a radioligand binding assay, suggesting that a serotonergic mechanism may be responsible for its anti-epileptic activity (Griffin et al., 2017). Interestingly, fenfluramine, an inducer of serotonin (5-hydroxytrypamine, 5-HT) release, which was found to have some efficacy in improving seizures in individuals with Dravet syndrome (Ceulemans et al., 2012), also reduced seizure activity in zebrafish *scn1lab* mutants and morphants, suggesting conservation of pharmacological pathways (Dinday and Baraban, 2015; Zhang et al., 2015; Sourbron et al., 2016).

Based on the serotonergic mechanism of fenfluramine, (Sourbron et al., 2016) tested selective 5-HT receptor agonists in *scn1lab* mutants and found that 5-HT_1D_, 5-HT_2C_, and 5-HT_2A_ agonists reverse electrographic seizure activity. Also, Griffin et al. (2017) screened a library of 5-HT-modulating compounds and found that lorcaserin and trazodone rescued seizure activity. Through a compassionate use program, lorcaserin was subsequently prescribed to five patients with Dravet syndrome with intractable seizures. While these patients experienced an initial decrease in seizure frequency, seizures returned to baseline after 3 months in most patients (Griffin et al., 2017). Clearly, larger, double-blind, placebo-controlled trials are needed to fully assess the efficacy of this medication. Another important consideration is how to accurately translate effective dosages between systems. Nonetheless, these studies highlight the strengths of zebrafish as a first-pass screening approach for identifying potential anti-epileptic drug candidates. Indeed, as more epilepsy-associated genes are identified in human studies, it is likely that zebrafish models will continue to be instrumental in this regard. For example, zebrafish mutants of *stxbp1b*, an ortholog of *STXBP1*, which is associated with epileptic encephalopathy syndromes, display electrographic seizures at baseline, suggesting this may be a useful model for these syndromes (Grone et al., 2016).

### Screening Risk Genes Associated With Epilepsy and Intellectual Disability

Zebrafish have also been used a genetic tool for rapidly screening the functionality of novel genes and rare variants identified in human genetics studies of epilepsy, ID and other neurodevelopmental disorders (Table 3, Bassuk et al., 2008; Gauthier et al., 2010; Suls et al., 2013; Schubert et al., 2014; Kury et al., 2017; Reijnders et al., 2017; Marin-Valencia et al., 2018). These studies assess the extent to which wild-type mRNA or mRNA carrying rare variants identified in affected individuals reverses morpholino-induced or CRISPR F0 phenotypes, providing an *in vivo* readout of the effect of the mutation on gene function. For example, morpholino-induced knockdown of *STX1B*, which was identified by linkage analysis in large pedigrees as carrying damaging mutations in individuals with epilepsy, caused electrographic seizures (Schubert et al., 2014). These seizures were reduced by CNS-specific expression of human *STX1B* mRNA, but not mRNA carrying a patient mutation, demonstrating that this variant represents a loss-of-function. In addition, morpholino-induced knockdown of *TRAPPC6B*, which was identified as a risk gene by linkage analysis and homozygosity mapping in individuals with epilepsy, microcephaly, and ASD from consanguineous families, led to increased baseline neural activity and sensitivity to PTZ-induced seizures in zebrafish larvae (Marin-Valencia et al., 2018).

Another approach to assess the functionality of newly identified human genes or rare variants is overexpression in zebrafish embryos. For example, overexpression of wild-type *pk1a*, the zebrafish ortholog of *PK1A*, caused a more severe phenotype than overexpressing mRNA carrying a mutation identified in individuals with progressive myoclonic epilepsy, suggesting that the mutation alters the *in vivo* function of this gene (Bassuk et al., 2008). In addition, overexpression of mRNA encoding human *RHEB* and versions of the gene containing two missense mutations identified in individuals with ID and macrocephaly, caused macrocephaly in zebrafish larvae, while F0 CRISPR mosaics of this gene displayed microcephaly, suggesting these variants may represent a gain-of-function (Reijnders et al., 2017).

There are several points to consider in the use of zebrafish for screening variants identified in human genetics studies. First, while *in vivo* rescue or overexpression screens may be informative regarding the biological function of an identified variant, the identification of nonspecific neural phenotypes, particularly morpholino-based phenotypes, are not sufficient to establish causation of an identified gene or variant and should not be used a substitute for strong evidence from human genetic studies. Second, while the presence of a phenotype in CRISPR F0 mosaics provides additional support for specificity, it is important to demonstrate in a stable mutant line that the phenotype results from loss of gene function and not nonspecific effects in F0-injected embryos. Third, with advances in CRISPR technology (Auer et al., 2014; Kimura et al., 2014), it will be increasingly feasible to rapidly generate not only loss-of-function mutations in a gene of interest, but “knock-in” models of specific patient mutations, which will be particularly informative given the pleiotropy of genes associated with neurodevelopmental disorders.

### Schizophrenia

Schizophrenia is a psychotic disorder characterized by hallucinations, delusions and disorganized thought processes or behavior, as well as diminished affect, energy and motivation, which severely impacts overall functioning (American Psychiatric Association, 2013). The genetics of schizophrenia are complex, with over 100 common variants identified by genome-wide association studies (GWAS; Schizophrenia Working Group of the Psychiatric Genomics Consortium, 2014), and rare damaging variants and CNVs contributing to risk according to a polygenic model (Walsh et al., 2008; Purcell et al., 2014). This genetic architecture complicates the functional analysis of risk variants in schizophrenia (Fromer et al., 2016). Here, we discuss several studies that used zebrafish to analyze the function of schizophrenia-associated genes (Table 4).

While schizophrenia is highly polygenic, *DISC1* is an example of a rare schizophrenia-associated gene, which was discovered in a large Scottish family where a balanced chromosomal translocation segregated with schizophrenia and other psychiatric disorders (schizoaffective disorder, bipolar disorder, major depressive disorder; Millar et al., 2000). Some studies have used zebrafish to investigate *DISC1* function. For example, De Rienzo et al. (2011) found that *disc1* mutants and morphants display abnormal brain morphology at early developmental stages, including small brain ventricles. Full-length human *DISC1* mRNA rescued structural brain phenotypes in morphants, while *DISC1* lacking the GSK3β binding domain did not, suggesting that that loss of Wnt signaling is responsible for these phenotypes. In a subsequent study, Singh et al. (2011) showed that two common variants in *DISC1* identified in individuals with schizophrenia and bipolar disorder that lacked Wnt signaling activity in an *in vitro* assay were unable to rescue structural brain phenotypes in *disc1* morphants, further implicating Wnt signaling as an important pathway downstream of *DISC1*. Another study found evidence for altered hypothalamic development as well as stress responses in zebrafish *disc1* mutants (Eachus et al., 2017). Interestingly, this study found variable phenotypes in two *disc1* mutant lines, such as differences in the time course of expression changes in markers of hypothalamic precursors, even though both lines carry mutations that induce early premature stop codons. This suggests that the specific location of a mutation may alter the expression of a phenotype in mutants. Also, while this study used one of the same mutants as the previous study, no morphological defects were identified in *disc1* homozygous mutants, suggesting that background variation may alter the expression of phenotypes in genetic mutants (Eachus et al., 2017). Together, these studies highlight insights into *DISC1* function gained from zebrafish models.

Additional studies have used zebrafish to rapidly assess the effect of changes in the expression of schizophrenia candidate genes implicated in human genetics studies. By comparing risk variants identified by GWAS with RNA sequencing data from post-mortem brain samples from individuals with schizophrenia, Fromer et al. (2016) identified genomic loci where risk variants might contribute to observed changes in gene expression. By altering the expression of three implicated genes in zebrafish in the same direction as the RNA sequencing result from human brain tissue, this study found that morpholino-induced knockdown of the downregulated gene, *furina*, led to microcephaly and decreased cell proliferation, which was rescued by introducing human *FURIN* mRNA, while overexpression of the upregulated genes, *tsnare* and *cntn4*, led to microcephaly and increased cell proliferation (Fromer et al., 2016). Another study combining GWAS and human gene expression data identified *MAPK3*, which is found in the 16p11.2 interval, as a schizophrenia susceptibility gene. Morpholino-induced knockdown of *mapk3* caused microcephaly, which was reversed by overexpression of human *KCTD13*, another gene in the 16p11.2 interval (Gusev et al., 2018). As discussed earlier, while nonspecific neural phenotypes induced by morpholinos, overexpression, or CRISPR F0 mosaics, may be suggestive of a functional effect, replication of these findings in a stable mutant line is necessary for validation.

## Future Directions: Functional Imaging of Neural Circuits

Most studies of zebrafish models of neurodevelopmental disorders to date have focused primarily on early morphological and simple behavioral phenotypes. However, recent advances in functional imaging are likely to transform these studies in the near future, allowing for the assessment of circuit-level phenotypes resulting from risk gene disruption. Progress in brain imaging is due in large part to the development of genetically-encoded calcium indicators (GECIs), such as GCaMP, which provide a rapid readout of activity at the level of a single neuron (Chen et al., 2013). GCaMP can be expressed transgenically in a subset of neurons or throughout the brain of larval zebrafish, which is an ideal system for monitoring neural activity. By harnessing advances in imaging technologies, including two-photon and light-sheet microscopy, a number of studies are beginning to dissect neural circuit mechanisms in the developing zebrafish brain (Ahrens et al., 2013; Portugues et al., 2014; Bianco and Engert, 2015; Dunn et al., 2016; Filosa et al., 2016; Naumann et al., 2016; Thompson et al., 2016). Together with the transparency and relative simplicity of the larval zebrafish brain, these technologies are likely to have considerable translational potential for revealing mechanisms by which the disruption of risk genes leads to alterations in signaling networks in the developing vertebrate brain, resulting in simple behavioral phenotypes.

For example, several studies have used two-photon microscopy to record brain activity in response to visually-evoked stimuli. Two-photon microscopy is a point-scanning method that provides excellent spatial resolution, but is more limited in its imaging speed (Keller and Ahrens, 2015). To image brain activity, zebrafish are immobilized in agarose, while visual stimuli are projected onto a screen to the side or below the fish while brain activity is recorded. Portugues et al. (2014) used this approach to investigate the circuitry underlying the OKR, a reflexive series of eye movements induced by a rotating drum of alternating light and dark stripes. By simultaneously recording brain activity, eye and tail movements during stimulus exposure, this study identified a stereotyped pattern of brain activity that occurs during the OKR, and found that activity in specific brain regions correlates with sensory or motor signals. In addition, Filosa et al. (2016) used two-photon imaging to interrogate the neural circuitry governing feeding behavior. Interestingly, this study showed that hunger not only makes zebrafish more likely to pursue visual stimuli that resemble their food, but increases the responsiveness of specific cells in the optic tectum to these food-like stimuli, providing a neural correlate for the observed behavior. Other studies have also used two-photon microscopy to examine behavioral circuits, such as those involved in prey capture, predator responses, responses to visual and olfactory stimuli, and the optomotor response, a reflexive behavior that occurs following a perceived change in whole-field motion (Dreosti et al., 2014; Bianco and Engert, 2015; Dunn et al., 2016; Naumann et al., 2016).

In addition, a growing number of studies have used light-sheet fluorescence microscopy for functional imaging of the zebrafish brain. Light-sheet microscopy, which uses a thin “sheet” of light to illuminate samples, offers superior speed over two-photon microscopy (Keller and Ahrens, 2015). For example, this method was used to successfully image over 80% of the neurons in the larval zebrafish brain in approximately 1.3 s (Ahrens et al., 2013). Given its speed, light-sheet imaging was used to perform continuous whole-brain activity recordings at baseline, revealing functional networks of correlated activity in the zebrafish brain (Ahrens et al., 2013). Light-sheet imaging has also been used to study brain activity following exposure to various stimuli. For example, Thompson et al. (2016) found that distinct clusters of neurons in the optic tectum respond to visual, auditory and water flow stimuli, and provide evidence for integration in the processing of these stimuli. One drawback of light-sheet imaging is the potential for retinal activation by the light “sheet” itself (Keller and Ahrens, 2015). Two-photon imaging offers an alternative in this regard, because it provides stimulation outside of the visible range of zebrafish. Another approach is to position multiple light sheets to avoid direct retinal stimulation (Vladimirov et al., 2014). Further, functional imaging in general generates considerably large datasets, which may be difficult to analyze, though computational algorithms have been developed to address this challenge (Keller and Ahrens, 2015).

At the same time, these functional imaging techniques are technically challenging, not high-throughput, and often require immobilizing the fish. To address these limitations, Randlett et al. (2015) developed a technique called mitogen-activated protein kinase (MAP)-mapping, in which fixed brain tissue is stained for phosphorylated extracellular-signaling-regulated kinase (pERK), a marker of active neurons, and then imaged using confocal microscopy. To identify regions of differential activity, images are mapped onto a zebrafish brain atlas (Z-Brain). This approach can be used to obtain a readout of whole-brain activity in freely moving zebrafish either at baseline or in response to a stimulus or drug (Randlett et al., 2015). Another method, developed by Lovett-Barron et al. (2017), called MultiMAP, combines two-photon imaging of zebrafish during exposure to visual stimuli with immunostaining for neuronal cell types. By integrating the functional imaging and immunostained datasets, this method allows for the identification of the specific cell types that were active during a behavioral task. Intriguingly, using this approach, (Lovett-Barron et al., 2017) identified the neuromodulatory cell types controlling alertness in zebrafish, and found that manipulation of related cell types in mice induces similar behavioral effects, providing remarkable evidence for conservation of behavioral circuits in fish and mammals (Lovett-Barron et al., 2017). Therefore, findings in zebrafish models of neurodevelopmental disorders are likely to have translational relevance for understanding related circuits in mammals. Together, these technologies offer considerable promise for illuminating circuit-level mechanisms in zebrafish models of neurodevelopmental disorders.

## Conclusion

Zebrafish have critical advantages as a model system for investigating the function of genes associated with neurodevelopmental disorders. A growing number of studies are beginning to capitalize on their unique features to illuminate neurobiological and pharmacological pathways underlying ASD, epilepsy, ID and schizophrenia. These studies have utilized the transparency, tractability and throughout of the zebrafish model to identify the effects of loss of risk gene function on the development of specific neuron populations, molecular pathways, and simple behaviors, all of which can be leveraged to screen for novel small molecule suppressors. While many studies to date have used morpholino knockdown technology, which is prone to off-target effects and should not be used as a “standalone tool” (Lawson, 2016), it is important moving forward that the field commit to using genetic mutants to confirm morpolino-induced phenotypes, which is particularly essential for neural phenotypes. While the advent of CRISPR technology has made this goal increasingly feasible, confirming that CRISPR-generated mutants result in loss of function is equally critical. Because splice-site mutations may lead to alternative transcripts that reverse deleterious mutations (Anderson et al., 2017), targeting CRISPRs within a conserved exon, removing most of a target gene using multiple CRISPRs, and demonstrating loss of protein by western blot are recommended steps.

At the same time, one of the challenges in analyzing the function of the growing list of risk genes associated with neurodevelopmental disorders is determining which phenotypes are likely to be relevant to the pathophysiology of these disorders (State and Šestan, 2012). Assessing phenotypes over a developmental time course from embryonic to adult stages will likely provide key insights into when and where risk genes play important roles. In addition, investigating the effect of specific mutations identified in affected individuals by introducing or “knocking in” these mutations using CRISPR will also be instrumental in elucidating how particular variants affect neural development. Moreover, future studies capitalizing on the strengths of zebrafish as a first-pass, high-throughput screening approach have the potential to reveal novel pharmacological candidates for further investigation in these disorders. Given the evidence for conservation of pharmacological and circuit-level pathways in zebrafish and mammals (Rihel et al., 2010; Lovett-Barron et al., 2017), it is likely that these studies will have translational relevance, though testing compounds identified in zebrafish in rodent models will be an important next step prior to clinical trials in humans. Furthermore, advances in *in vivo* calcium imaging in zebrafish represent an exciting new avenue for investigating the circuit-level roles of risk genes with translational relevance. Taken together, zebrafish represent a promising model system for the discovery of novel biological pathways, pharmacological candidates, and circuit mechanisms with relevance to neurodevelopmental disorders.

## Author Contributions

CS, SI and EH contributed to the conceptualization, literature review and writing of this article.

## Conflict of Interest Statement

The authors declare that the research was conducted in the absence of any commercial or financial relationships that could be construed as a potential conflict of interest.
